# The right to die: Perspectives of mental health professionals in malta

**DOI:** 10.1192/j.eurpsy.2021.1001

**Published:** 2021-08-13

**Authors:** G.J. Ellul

**Affiliations:** Mental Health Department, Mount Carmel Hospital, Attard, Malta

**Keywords:** ethics, Euthanasia, AssistedSuicide, psychiatry

## Abstract

**Introduction:**

In their professional work, mental health professionals are continually working with individuals in distress, who may express a wish to end their lives.

**Objectives:**

To understand the perspectives of mental health professionals towards a person’s right to die.

**Methods:**

A mixed-method technique was used: Stage 1 involved a validated online questionnaire sent to all professionals working within the public mental health services in Malta. Stage 2 consisted of a multidisciplinary discussion between six professionals asked to hypothetically manage a terminally ill patient requesting physician-assisted suicide. Thematic analysis was subsequently applied.

**Results:**

The majority of mental health professionals disagreed with allowing a person to commit suicide, even in situations of crippling debt, overwhelming despair and family dishonour. Terminal illness elicited a varied response (Figure 1)

**Figure:**
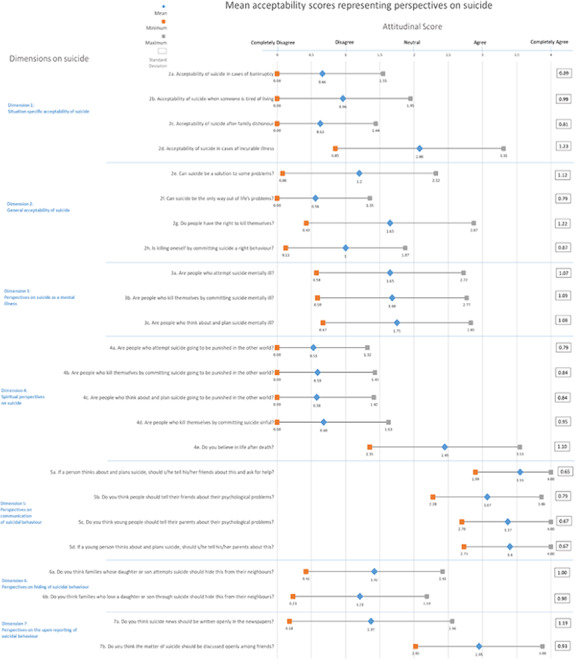

Older professionals and spiritual beliefs negatively impacted acceptability of suicide (Figure 2).

**Figure:**
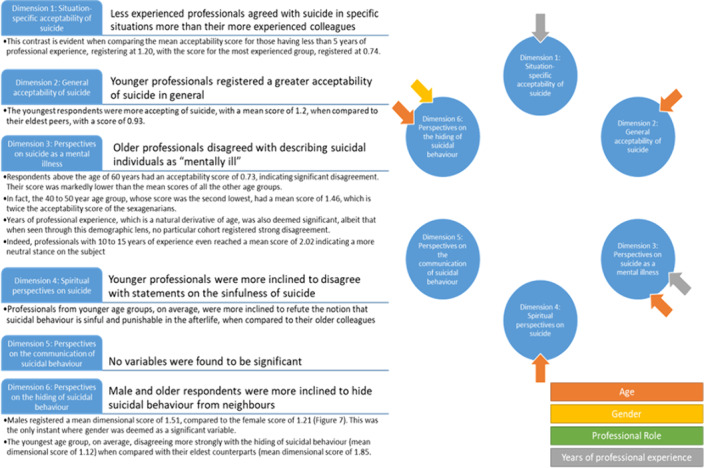

The discussion revealed that professionals would assess individuals requesting to end their lives, with the aim of treating any mental illness and determining mental capacity. Figure 3 highlights factors explored during the assessment. Greatest emphasis is ultimately placed on individual autonomy.

**Figure:**
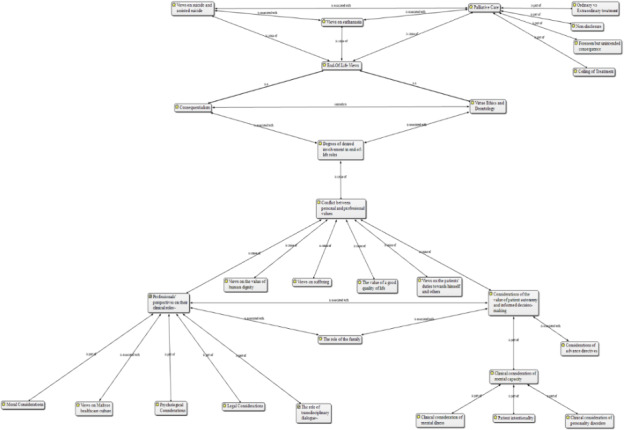

**Conclusions:**

Mental health professionals consider autonomy and self-determination as imperative in evaluating a person’s right to die. Professionals agreed that, after a comprehensive psychiatric assessment and within a regulatory legal framework, they would not impede a person with terminal illness to request physician-assisted suicide, provided that one is acting autonomously. The majority would however conscientiously object to actively assisting the terminal patient in ending one’s life, since this is deemed contradictory to their professional vow of non-maleficence.

